# Cognitive-Behavioral Play Therapy and COVID-19 Pandemic Trauma in Preschool Children

**DOI:** 10.7759/cureus.44249

**Published:** 2023-08-28

**Authors:** Jaroslava Raudenska, Jiří Gumančík, Martin Raudenský, Alberto Pasqualucci, Eleni Moka, Giustino Varrassi, Antonella Paladini, Alena Javurkova

**Affiliations:** 1 Department of Nursing, Second Medical Faculty of Charles University, Prague, CZE; 2 Department of Psychology, Faculty of Health and Life Sciences, University of Northumbria in Newcastle, Newcastle upon Tyne, GBR; 3 Faculty of Education, Charles University, Prague, CZE; 4 Department of Anesthesia and Critical Care, University of Perugia, Perugia, ITA; 5 Department of Anesthesiology, Creta InterClinic, Heraklion, GRC; 6 Department of Pain Medicine, Paolo Procacci Foundation, Rome, ITA; 7 Department of Life, Health and Environmental Sciences (MESVA), University of L'Aquila, L'Aquila, ITA; 8 Department of Clinical Psychology, University Hospital Královské Vinohrady, Prague, CZE; 9 Department of Nursing, Second Medical Faculty of Charles University and Motol University Hospital, Prague, CZE

**Keywords:** preschool age, cognitive-behavioral play therapy, play, covid-19 pandemic, trauma

## Abstract

Traumatic life events, such as the ongoing COVID-19 pandemic, can be a challenging health emergency, among others. The pandemic can also affect young preschoolers. They can experience negative emotions and behavioral and social difficulties. It can be hard for children to understand on their own what is going on, as well as for their families. This narrative review summarizes the role of psychotherapeutic approaches in working with trauma and aims to manage trauma, such as COVID-19, in preschool children. It focuses on the possibilities of applying cognitive-behavioral play therapy (CBPT) in preschool children who suffer from trauma during the COVID-19 pandemic period. The therapeutic approaches are tailored to specific development in preschoolers. Furthermore, the article provides insights into the relationship between negative emotions, thinking, and behavior, and COVID-19 as a threat, by describing a cognitive model in preschool-aged children. Finally, the article offers possible ways of applying play-based cognitive-behavioral therapy programs in preschool children who are victims of trauma.

## Introduction and background

Trauma can be an injury, a potential threat of death, or a threat to the integrity of oneself or others, and it can be described by the presence of events, experiences, and effects [1]. A traumatic life event usually causes horror, terror, or helplessness at the time of occurrence. It can include physical, emotional, sexual, domestic, community, school, or workplace violence. Traumatic life events also include medical trauma, acute accidents, war or terroristic attack experiences, catastrophes, social crises, discrimination in close family relationships, and other related traumatic losses [2,3].

Traumatized children can develop emotional or behavioral problems following traumatic experiences [4-7]. Trauma influences physical and mental health in short- and long-term points of view [3]. In the short term, trauma can be associated with mental health problems such as anxiety, depression, post-traumatic stress disorder (PTSD), dissociation, depersonalization, and/or emotional dysregulation. In the long term point of view, trauma can cause physical (somatization, pain, and alterations of the immune system), social, and intellectual problems [8-10].

Experiencing a pandemic, such as the COVID-19 pandemic, may be also characterized as a mass traumatic event. In turn, this experience might guide further trauma, trauma responses, or traumatic life events, which are related to COVID-19 [1,11-16]. The mental health impact of COVID-19-related stressors is described as much more severe than other traumatic life events [17]. Exposure to multiple traumatic events related to the COVID-19 pandemic can result in long-term negative effects on children [3], particularly on many aspects of the child’s emotional, social, cognitive, behavioral, and future development, including the formation of a sense of self. However, there are mixed opinions when it comes to diagnosing someone who has experienced trauma. It is possible to define the COVID-19 global pandemic as a traumatic event according to the Diagnostic and Statistical Manual of Mental Disorders, Fifth Edition [18,19], or stress associated with the COVID-19 pandemic can be diagnosed differently with adjustment disorder or diagnosis of other specified trauma and stressor-related disorder [18-21]. However, the situation with preschool children may be different from that of adults.

## Review

Aim

The aim of the article was to apply the model of cognitive-behavioral play therapy (CBPT) in managing trauma in connection with the COVID-19 pandemic.

Search Strategy, Selection Criteria, and Results

We searched for relevant sources in PubMed, Scopus, and Web of Science databases and additional searches through Google Scholar from the past 25 years to June 2023 using the following keywords: “play,” “play therapy,” “children,” “preschool age,” “cognitive-behavioral therapy,” “cognitive-behavioral play therapy,” “psychotherapy,” and “trauma.” For searches from 2020 to 2023, the keyword “COVID-19” was added. The search on PubMed using “play,” “cognitive,” “behavioral,” and “therapy” resulted in 158 publications. Using “play,” “children,” “cognitive,” “behavior,” “therapy,” and “trauma, only 45 papers were retrieved. There were no results using the keywords “play,” “preschool,” “cognitive,” “behavioral therapy,” “trauma,” and “COVID-19” in the period 2020-2023. Using only “preschool,” “trauma,” and “COVID-19” in the same period of time, we retrieved 23 publications.

The same search was repeated in the other two databases, Scopus and Web of Science, but again, we did not find too many relevant sources/literature. We did an additional search through Google Scholar, especially in books and book chapters. We used the keywords “play,” “play therapy,” “children,” “preschool,” “cognitive-behavioral therapy,” “cognitive-behavioral play therapy,” “psychotherapy,” and “trauma.” For searches from 2020 to 2023, we added the keyword “COVID-19.”

Articles of different designs in full text, book chapters, and books in the English language were included. All retrieved sources were screened by title, abstract, and full article/chapter/book itself. The sources had to be related to play, preschool age, CBPT, cognitive and cognitive behavioral therapy, trauma, and COVID-19.

A total of 237 eligible sources were retrieved from PubMed, 210 from Scopus, and 115 from Web of Science. The number of additional sources identified through Google Scholar was 119. We screened 681 sources. When duplicates from all databases had been removed, a total of 599 titles and abstracts were screened. We excluded 515 sources (sources that did not assess children, play therapy, cognitive-behavioral therapy, trauma, or the COVID-19 pandemic). Finally, we included 84 sources, which will be illustrated and discussed in this narrative review article.

COVID-19 pandemic and psychological development of preschoolers

Preschool development is the result of attachment relationships, cognitive and speaking abilities, and regulation of emotion in the context of family, society, and culture [12]. People differ in their genetics, development, health, resilience, support, and social and cultural background. Preschool children’s mental health is shaped from the earliest days. It is a mixture of genetic factors, environmental factors, parenting, quality of close relationships, and exposure to trauma and challenging health emergencies such as the COVID-19 pandemic. Both adults and children experienced the COVID-19 pandemic. For many children, the short-term adverse effects and their impact on their well-being passed. For some, problems started. To understand the trauma related to the COVID-19 pandemic, cultural differences and developmental issues have to be considered. Especially in preschool children aged 2-6 years, because of their specific cognitive, behavioral, and emotional development, complex trauma related to the COVID-19 pandemic can be manifested on the somatic level (belly pain, headache, stomachache, toothache, and other somatization) or behavioral level. This is the so-called “angry behavior” or anxious behavior. Each child may behave in a typical or nontypical way. Preschool children can often feel helpless; they do not want to be separated from adults, scream or cry a lot, lose their appetite, can often have nightmares, use baby talk, reuse diapers, and recreate the trauma through play and play activities [3,10,22]. Not all preschool children exposed to trauma will develop negative symptoms of short- and long-term mental health consequences [23]. Only a small group with relevant effects of traumatic life events can be identified as suffering from trauma [1]. These findings open up the topic of working with children through matters such as negative emotions, cognition, stress, and/or behavioral troubles. This is especially important during the COVID-19 pandemic, as it brings recurring lockdowns and other related problems.

Negative Consequences of the COVID-19 Pandemic Lockdown

People were obliged to follow specific public health instructions during the pandemic, limiting personal freedom. This is the reason why the COVID-19 pandemic and its consequences may be traumatic, especially for preschool children. Since February 2020, when the pandemic started, 14% of American parents reported that their children’s behaviors had worsened and that they were not emotionally stable [20,24]. Negative emotions were formed as new virus variances and illnesses related to them appeared. These emotions were particularly strong toward social distancing measures and their consequences. Moreover, lockdowns, the inability to attend preschool activities, the lack of any social interactions with peers, long-term experience of fear of possible illness, and other threats significantly affected the behavior of preschool children. Several studies suggested a tendency toward adverse consequences on children’s emotional and behavioral function in relation to lockdowns during the COVID-19 peak [11,12,15-17,25-28]. Moreover, during the time when there was no lockdown, the threat of COVID-19 disease was ubiquitous. Hence, this is why the study by Idoiaga et al. [29] decided to explore the relationship between the pandemic measures, children’s emotional representation of the COVID-19 crisis, and their coping ability with its adverse effects. Other studies found that preschoolers experienced difficulties in understanding the causality of COVID-19 infection [15]. Furthermore, they expressed worries about getting sick and infecting others, which was reflected in their play and drawings. Additionally, they also showed difficulties in attachment-seeking behavior [29]. On the contrary, one study demonstrated improvement in outcomes of preschool wheezers, with a reduction of respiratory symptoms, medication use for exacerbations, and use of healthcare resources during a lockdown, but the authors believed that these outcomes would reverse when the lockdown is lifted [30]. Although it seemed that staying at home was positive for children in the last described study, many children were at risk of complex traumas [31]. Staying at home and not attending preschool activities can induce trauma and social isolation. This it is also associated with physical and sexual abuse and neglect, violence, witnessing violence between parents or siblings, financial problems of the family, and other difficulties [8,32]. Therefore, collecting data to examine mental health outcomes of young children (1-5 years) during the pandemic is important [12,33]. It is also equally important to identify and treat depression, anxiety, and post-traumatic stress disorders in preschool children at the earliest.

Psychotherapy in Children After Trauma Exposure

Psychotherapy has an important role in children’s coping when traumatic life events and trauma-related difficulties occur [7,20,34]. The challenges of psychotherapy that are focused on young children include the assessment and choice of the best therapeutic strategy. Psychotherapeutic approaches for young preschool children are developmentally specific. Cultural differences have to be considered to understand trauma and to know the best type of assessment, treatment, and psychotherapy to use [6].

Trauma-focused cognitive behavioral therapy (TF-CBT) is an evidence-based psychotherapy for coping with trauma in adults, adolescents, and young children [35-37]. Other programs in children and adolescents include client-centered play therapy, eye movement desensitization and reprocessing (EMDR) [23], kid narrative exposure therapy (KIDNET) [38], Support for Students Exposed to Trauma (SSET) [39], and Trauma Grief Component Treatment (TGCT) [40]; however, these programs are still experimental. It is clear that evidence-based approaches for preschoolers and evidence-based psychotherapy for coping with trauma in preschool children are not too broad (child-parent psychotherapy (CPP) [41], TF-CBT [4,36,42], and play therapy). Meta-analyzes and systematic reviews, RCTs, quantitative and experimental surveys, and qualitative studies support the use of play therapy in children aged 3-12 years [43]. Play therapy may include a variety of orientations and specific protocols, such as child-centered or cognitive-behavioral play therapy (CBPT), gestalt psychology, or Adlerian protocols [43-45]. CBPT is rooted in the evidence-based theory of CBT [43,44]. Professional therapists need to be trained and need to continuously undergo long-term supervision for CBPT and trauma therapy [46]. CBPT should not be overlooked in regard to coping with the complex traumatization of preschoolers due to the COVID-19 pandemic.

Cognitive-behavioral play therapy (CBPT)

For children of preschool age, playing is a very natural activity that represents a universal expression of childhood by using fantasy, symbols, and imagination. Through play, relationships with adults and other people are established naturally; children learn to control urges, stimuli, and stress, as well as social skills. Play may allow children to experience a sense of power and control when solving problems and coping with new ideas and interests by providing a sense of certainty, courage, and success. Through play and play-based interventions, children can communicate symbolically and in an action-oriented manner. Trained psychotherapists use the therapeutic aspects of play to help children prevent or resolve psychosocial problems and reach optimal growth and development [43,47]. Therefore, play therapy is one of the major instruments of counseling and psychotherapy in preschool children. Additionally, it is an effective intervention for children who show reactions to traumatic events.

Nonetheless, as early as the mid-1980s, cognitive-behavioral (CB) techniques and cognitive therapy began to be incorporated into play therapy, with regard to the child’s developmental level within the population of younger preschool-age children [48,49]. This occurred mainly during the 1990s [50-52]. Hence, CBPT was developed, adapting adult principles of CBT [53] to children’s developmental requirements [43,44,54-56].

In traumatized preschool children, it is important to integrate CBT with play techniques. Cognitive strategies can be used in children if adapted to the child’s age and needs. Utilizing puppets, dolls, figures, and other materials is suitable when demonstrating the modeling technique and practicing alternative ways of behavior and problem-solving using role-play. It is goal-oriented and focused on cooperation with patients and their families. In addition, this CBPT technique contains the components of traditional non-directive play therapy: trust within the therapeutic relationship and play as communication between the therapist and the child. However, there are differences in the conduct, therapeutic goals, selection of play material, and activities by therapists, as well as children, play as psycho-education and the therapist as a coach (showing children the relationship between their behaviors and thoughts) [51].

CBPT has been effectively used in children impacted by mental health problems such as sexual abuse [57,58], trauma, and domestic violence and children with weak development of social skills, emotional disorders [59,60], aggression [61], attention deficit hyperactivity disorder [62], and pain [59,63-64]. Trauma-focused integrative play therapy is an example of an integrative approach, using selected toys to serve as models and symbols of trauma [65,66]. A play that works with post-traumatic problems has goals similar to those of the CBT technique. CBPT may be used in anxious children to rehearse self-regulatory behaviors, such as coping with medical procedures, or systematic desensitization in phobias [59]. It is also useful in medically ill and obese children who usually suffer in school during certain social situations and/or relationships with others [67]. The COVID-19 pandemic as a trauma is a challenge in clinical settings.

Cognitive play model

The Connection Between Thoughts, Feelings, and Behaviors in Preschoolers

A cognitive model, recognition of negative thoughts and worries, explains the development and maintenance of anxiety, depression, and post-traumatic stress following traumatic life events. Preschool children are at the preoperational stage of cognitive development. A therapist is interested in the impact of maladaptive or dysfunctional beliefs on the attitudes and behaviors of the child. In addition, they also focus on how their reactions to events are influenced by their subjective perceptions. Egocentrism, the concrete way of thinking, and irrational thoughts of a preschool child exclude the cognitive skills necessary to take part in cognitive therapy.

**Figure 1:**
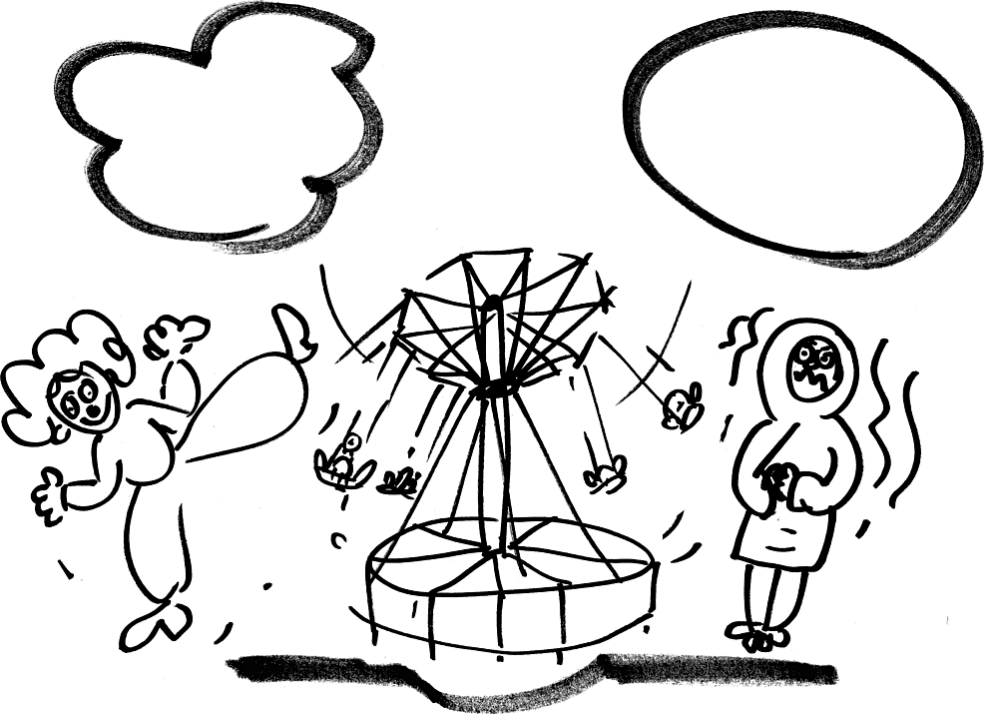
Drawing of a therapist Author’s (MR) personal collection

Helping children to understand the connection between thoughts, feelings, and behaviors is a key step in teaching them how to examine and exchange cognition via play. The cognitive model can be explained using a story, which can be modified as needed [68]. The story should be presented at the beginning of therapy, usually during the first or second session. To better understand the model, therapists can also draw pictures on paper or a whiteboard, or draw illustrations, while telling the story. An example of this is the story about the carousel in the park and two children, one of them fearful to play and the second happy to play in the carousel (Figure 1).

**Figure 2:**
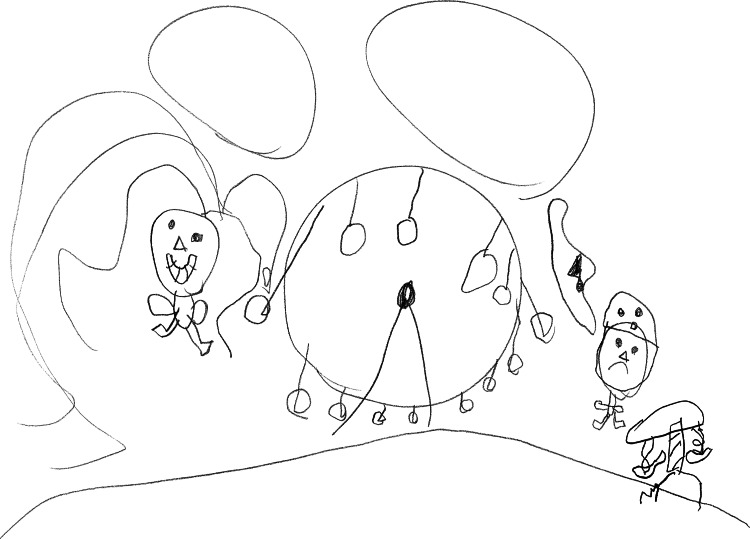
Drawing of a five-year-old boy Author’s (MR) personal collection

That may be a well-known situation for children. The model can also be used in a way where the therapist asks the child to draw the picture himself (Figure 2). It is very important to find a way to present a story that is comfortable, both for the child and the therapist; the therapist needs to use language that is appropriate and comprehensible to the preschool child. The drawing will help to understand the relationship between emotions, thoughts, and behavior, for example, an anxious child who is afraid to play on the carousel so as not to get hurt and a child who is not afraid to play on the carousel. Bubbles, similar to those in comics, for each child in the picture give the opportunity to find out what the child thinks and how he/she feels. In this way, the cognitive model is explained to preschool children. Possible changes are communicated in therapy indirectly through the use of play.

Cognitive Play Model Related to the COVID-19 Pandemic

We can effectively use this cognitive model to explain the relationship between negative emotions and, e.g., thinking related to the threat of COVID-19 in preschool children. A preschool child’s understanding of illness is associated with a child’s cognitive development. Preschoolers aged 2-6 years are still in the preoperational stage of thinking. They are unable to explain illness and what causes illness, especially COVID-19. They can see the reason for the illness, mostly external reasons. Sometimes, reasons can be magical but also unexplainable. Children at this age can see the virus as an enemy and can be fearful and worried that they could transmit the virus to other members of the family, particularly to grandparents [28].

Therefore, materializing, e.g., drawing the COVID-19 virus, or another related problem, and trying to explain to the child how our emotions, thoughts, and behaviors are connected can be a good application of CBPT in the current situation or in other traumatic circumstances. Education, desensitization [69], modeling [70], generalization, and relapse prevention are used as interventions within the framework of CBPT. Education can help children to understand what is going on and to explain all worries with regard to age. Models of puppets and soft toys can be used by employing problem-solving skills. The child acts out traumatic situations related to COVID-19, acquiring mastery over them. Experiments can be performed by the therapist who will help the child engage in cognitive therapy. During play therapy, children should be given the opportunity to express their feelings of fear using pictures, stories, and role-play with puppets. Later, the therapist can help the child experiment in an interview about the fear of the puppets who were afraid of some situations related to COVID-19 and other traumas. The interviews should include a list of fears and positive coping statements (“I can think of something nice, which will help me to be satisfied,” “I can think of mum/dad who always comes back for me”). Through the puppet’s voice, the therapist creates adaptive coping skills for the child. The children will gradually incorporate their own stories and puppet play, finally applying them to their own behavior. Socratic interview is usually modified for use with preschool children [71]. New information may be used adaptively through modeling with play material and using more open-closed questions (e.g., “I would like to know what you think about the lockdown related to the COVID-19 pandemic” rather than “What do you think about the lockdown related to the COVID-19 pandemic?”), enabling the child to experience the therapist’s interest.

Evidence-based CBPT programs

The first implementation of CBPT in prevention was described several years ago [72]. Specific programs, using CBPT in preschoolers and young children, can be an inspiration for integrating them into work with traumatized preschoolers during the COVID-19 pandemic and other stressful situations.

Coping Cat Program

This program consists of 16 therapy sessions based on CBT, with regard to the child’s developmental stage [73,74]. It focuses on coping with generalized or separation anxiety and social phobia. It blends CBT and play, amusing activities, drawing, cartoons, and emotional scales. The program is suitable for children aged 5-13 years and can be administered in individual or group sessions. A workbook [75] and a separate therapist manual [76] are available in English. The manual provides an overview of the general strategies used in the treatment of anxiety disorders in children. It also provides strategies for dealing with potential difficulties in therapy, such as noncompliance. The program is also available in a version for adolescents aged 14-17 years [77]. There are also modifications to the program, especially for anxious children [78-81].

Parent-Child Interaction Therapy (PCIT)

This program originally consisted of 20 behavioral sessions for parents with children affected by disruptive behavior disorders and aged 2-8 [82,83]. It is composed of two phases. The first part consists of a child-directed interaction that enriches the parent-child relationship by using aspects drawing on filial therapy. Parents encourage their children during play situations to increase positive parenting and warmth in the parent-child interaction. The second phase is parent-directed and consists of behavioral therapy. Parents are coached by therapists to use behavioral techniques in connection with play, including behavior reinforcement through rewards and praise. Meta-analysis showed that PCIT has positive outcomes on parent-reported and parent-child interaction measures in different children populations [84,85].

Coping Power Program

This program was developed on the basis of a preventive intervention for anger management: the Anger Coping Program. It has been extended to include interventions, target children, and focus on parent training in behavioral techniques. This intervention is intended for children with aggressive behavior and their parents, being delivered to both groups during the same period of time in 16 group sessions. Usually, the program is delivered to children in late elementary to early middle school years; for preschoolers, this has to be modified. The program is effective in preventing anger management issues [86]. Parents use a manual and workbooks [87-89]. Structured play techniques, modeling, and role-playing are used. The therapist manual describes step-by-step instructions for accurately implementing the program. The corresponding workbook for children includes worksheets and monitoring forms for tracking progress and success and reinforcing the skills learned in the group sessions [90]. For preschoolers, this has to be modified as well. A recent systematic literature review showed that it effectively reduces negative behavior, but a meta-analysis showed small reductions in maladaptive behavior, with no significant findings [91].

Kids Together Program

This consists of a 15-week group play therapy program for children aged 5-16 years with attention deficit hyperactivity disorder (ADHD). They often experience impulsivity, aggressive behavior, or lack of social and communication skills [92]. Therefore, the program is targeted primarily at improving their social relations. Therapy uses a combination of play, arts, and leisure activities. Social skills training is carried out using emotional identification, communication, conflict-solving, and relaxation. The model is psycho-educative and based on play therapy. A recent study showed that it may be adapted for children with disabilities [93].

Strengths and limitations

The strength of our work is to show the need to provide age-specific and development-friendly psychotherapy using CBPT in preschool children traumatized by the COVID-19 pandemic or other psychological traumas. CBT for preschool children has been associated with play as a basic means of expression at this age. This is significantly positive for this age group.

Another positive thing about our work is that we can already use the knowledge from well-established play-based CBT and CBPT programs. Therefore, we can target these lessons to set up programs to manage any psychological trauma, including the COVID-19 pandemic.

We must also mention some limits of this research. We only used publications in English, excluding other languages that would have probably provided further data. Another limitation is represented by the focus on only one therapeutic approach of play therapy, CBPT, in managing the trauma associated with the COVID-19 pandemic. Although it is evident that CBPT is an evidence-based approach to managing the trauma of preschool children, there are no clinical studies on the use of this program in managing the trauma of the COVID-19 pandemic. Although the CBPT literature on traumatic events is extensive, the COVID-19 pandemic has specific challenges. The individualized and culturally specific approach to managing COVID-19 trauma will also need to be adapted to these challenges in CBPT in preschool children.

## Conclusions

The COVID-19 pandemic is considered a mass, stressful, disruptive traumatic event. Trauma can have negative effects on preschool children’s mental health. Preschoolers’ developmental cognitive stage does not allow for the understanding of the causality related to the COVID-19 pandemic. CBPT is an appropriate therapy that focuses on coping with trauma and is based on the evidence of CBT in preschoolers. It can be used in preschool and traumatized children who suffer from a complex trauma connected to the pandemic. Cognitive models and play techniques form a more adaptive view of a child’s self, the world, the future, and the place of the child in it. CBPT also helps to promote children’s social and emotional competence and builds positive relationships. Early enough, preventive, and therapeutic interventions aimed at emotional, cognitive, and behavioral troubles related to COVID-19 pandemic trauma are more effective when done at preschool age, rather than later or never. CBPT should not be overlooked during the COVID-19 pandemic, as the pandemic may generate complex traumatization of preschoolers due to their specific developmental approach. However, more research and understanding are needed in the field of CBPT applications in children affected by psychological trauma post-COVID-19 pandemic.
